# Microvascular reactivity and clinical outcomes in cardiac surgery

**DOI:** 10.1186/s13054-015-1025-3

**Published:** 2015-09-04

**Authors:** Tae Kyong Kim, Youn Joung Cho, Jeong Jin Min, John M. Murkin, Jae-Hyon Bahk, Deok Man Hong, Yunseok Jeon

**Affiliations:** Department of Anesthesiology and Pain Medicine, Seoul National University Hospital, 101, Daehak-Ro, Jongno-Gu, 03080 Seoul, Korea; Department of Anesthesiology and Pain Medicine, Samsung Medical Center, 81, Irwon-Ro, Gangnam-Gu, 06351 Seoul, Korea; Department of Anesthesiology and Perioperative Medicine, Schulich School of Medicine, University of Western Ontario, 4, 1465 Richmond St, N6G 2M1 London, ON Canada

## Abstract

**Introduction:**

Microvascular reactivity is decreased in patients with septic shock; this is associated with worse clinical outcomes. The objectives of the present study were to investigate microvascular reactivity in cardiac surgery patients and to assess any association with clinical outcomes.

**Methods:**

We retrospectively analyzed a prospectively collected registry. In total, 254 consecutive adult patients undergoing cardiac and thoracic aortic surgeries from January 2013 through May 2014 were analyzed. We performed a vascular occlusion test (VOT) by using near-infrared spectroscopy to measure microvascular reactivity. VOT was performed three times per patient: prior to the induction of anesthesia, at the end of surgery, and on postoperative day 1. The primary endpoint was a composite of major adverse complications, including death, myocardial infarction, acute kidney injury, acute respiratory distress syndrome, and persistent cardiogenic shock.

**Results:**

VOT recovery slope decreased during the surgery. VOT recovery slope on postoperative day 1 was significantly lower in patients with composite complications than those without (3.1 ± 1.6 versus 4.0 ± 1.5 %/s, *P* = 0.001), although conventional hemodynamic values, such as cardiac output and blood pressure, did not differ between the groups. On multivariable regression and linear analyses, low VOT recovery slope on postoperative day 1 was associated with increases of composite complications (odds ratio 0.742; 95 % confidence interval (CI) 0.584 to 0.943; *P =* 0.015) and hospital length of stay (regression coefficient (B) −1.276; 95 % CI −2.440 to −0.112; *P =* 0.032).

**Conclusion:**

Microvascular reactivity largely recovered on postoperative day 1 in the patients without composite complications, but this restoration was attenuated in patients with composite complications.

**Trial registration:**

ClinicalTrials.gov NCT01713192. Registered 22 October 2012.

**Electronic supplementary material:**

The online version of this article (doi:10.1186/s13054-015-1025-3) contains supplementary material, which is available to authorized users.

## Introduction

Tissue hypoperfusion is one of the earliest warning signs in the critically ill [1]. It is considered a predictor of organ ischemia and postoperative clinical outcomes [2–4]. It is known that tissue hypoperfusion can occur with normal or even supranormal cardiac output due to impaired microcirculation [5]. The microcirculation is decreased in patients with sepsis [2], patients with heart failure [6], and critically ill patients [7]. Also, microcirculation is decreased in cardiac surgery patients, regardless of whether cardiopulmonary bypass (CPB) was used [8].

The vascular occlusion test (VOT) is a provocative test of the microcirculation, which uses the dynamic response of tissue oxygen saturation (StO_2_) to transient limb ischemia and reperfusion. StO_2_ is measured by near-infrared spectroscopy (NIRS), which is a real-time non-invasive indicator that reflects the ratio of oxygenated hemoglobin to total hemoglobin in the tissue. StO_2_ provides information about local tissue oxygenation and the state of the tissue microcirculation [9]. The recovery slope of StO_2_ during the VOT is used as a measure of microvascular reactivity because it reflects post-ischemic reperfusion and hyperemia [10, 11].

Previous studies have reported that microvascular reactivity is related to worse clinical outcomes in patients with sepsis [10, 12]. However, there are few data about microvascular reactivity during the perioperative period. Cardiac surgery, especially under CPB, is among the strongest inducers of inflammatory reactions [13] and in certain respects displays similarities with patients with sepsis. Thus, we hypothesized that decreased microvascular reactivity, measured by recovery slope, would be associated with worse clinical outcomes in cardiac surgery patients. The aims of the study were to investigate microvascular reactivity in cardiac surgery patients and to assess any association with clinical outcomes.

## Methods

### Patients

This was a retrospective analysis of a prospectively collected registry to assess microvascular reactivity in patients undergoing cardiac surgery. This study used data from the heart surgery registry at Seoul National University Hospital. It was approved by the institutional review board of Seoul National University Hospital, Seoul, Korea (institutional review board #1207-111-419) and was conducted in accordance with the Declaration of Helsinki. Written informed consent was obtained from each participant, and the trial was registered at ClinicalTrials.gov (NCT01713192). The registry enrolled all consecutive patients undergoing cardiac and thoracic aortic surgeries at Seoul National University Hospital from January 2013 to May 2014. The registry used a combination of techniques for quality control: standardized disease definitions and sampling techniques, clear entry on the data sheet, and review of cases by the managing physicians [14]. The registry included perioperative data with intraoperative hemodynamics, VOT data, and clinical outcomes. VOT was performed at designated time points, as part of the registry. For this study, we included patients from the registry in whom the VOT was performed. Patients who may not tolerate the VOT because of, for example, arm deformities, burns, arteriovenous shunts, and peripheral vascular disease, were excluded from analysis.

### Anesthesia and cardiopulmonary bypass techniques

All patients received standard perioperative care. Routine monitoring included a bispectral index, cerebral oximetry, pulmonary artery catheter, and transesophageal echocardiography. Anesthesia was induced with intravenous midazolam, sufentanil, and vecuronium. Anesthesia was maintained with continuous infusions of remifentanil (0.5–1.0 μg/kg per min) and propofol (0.04–0.07 mg/kg per min), targeting bispectral index values of between 40 and 60. Vecuronium was used as a neuromuscular-blocking drug. After tracheal intubation, lungs were ventilated mechanically, and ventilation was adjusted to maintain an end tidal carbon dioxide tension of 30–35 mm Hg.

In on-pump surgeries, a non-pulsatile CPB technique was used with a membrane oxygenator and cardiotomy suction. Cardiac protection was achieved by using antegrade/retrograde cold blood cardioplegia. Heparin was administered before CPB or coronary anastomoses and was neutralized with protamine after discontinuing CPB or completion of anastomoses. The target activated clotting times during surgery were more than 500 s for on-pump cardiac surgery and more than 300 s for off-pump coronary bypass graft surgery. At the end of surgery, patients were transferred to the intensive care unit (ICU).

Intraoperative treatment was standardized according to the routine protocol of our institution. The goal of hemodynamic management was to maintain a mean arterial pressure of 60-80 mm Hg, a cardiac index of more than 2.0 l/min per m^2^, and mixed venous oxygen saturation of more than 60 %. The decision of whether to administer additional vasopressors or inotropic drugs was taken by the attending physicians on the basis of the analyzed hemodynamic status.

### Vascular occlusion test

StO_2_ was measured continuously by using the InSpectra StO_2_ tissue oxygenation monitor model 650 (Hutchinson Technology Inc., Hutchinson, MN, USA). An NIRS sensor probe was placed on the thenar eminence and maintained in the same position during all measurements. If the probe was not attached properly to the thenar eminence, it was reattached before the VOT. The VOT was performed as described previously [11]. A conventional pneumatic blood pressure cuff was placed around the upper arm and inflated rapidly to 50 mm Hg above systolic blood pressure and remained inflated until the StO_2_ decreased to 40 %. Then, the cuff was deflated rapidly.

The VOT was performed three times per patient: prior to the induction of anesthesia, at the end of surgery (just before anesthesia cessation), and on postoperative day 1 (at 7 a.m. the next morning). The StO_2_ values were recorded continuously on the StO_2_ monitoring device at 2-s intervals and were analyzed by using the InSpectra Analysis software (version 4.03). The baseline StO_2_, and the VOT-derived occlusion and recovery phase parameters were recorded. Occlusion slope was determined by the decreasing line of the StO_2_ graph during pneumatic cuff inflation. The recovery slope was determined by the increasing line of StO_2_ graph after pneumatic cuff deflation.

### Study outcomes

The primary endpoint was a composite of major complications that included in-hospital death, myocardial infarction, acute kidney injury, acute respiratory distress syndrome, and persistent cardiogenic shock. The definition of each major complication was as follows. Myocardial infarction is defined as elevation of cardiac biomarker values (>10× 99th percentile upper reference limit) in patients with normal baseline troponin values (<99th percentile upper reference limit). Additionally, new pathological Q waves or new left bundle branch block, or angiographically documented new graft or new native coronary artery occlusion, or imaging evidence of new loss of viable myocardium or new regional wall motion abnormality was required [15]. Acute kidney injury was defined according to the Risk, Injury, Failure, Loss, and End-Stage Kidney Disease (RIFLE) criteria (Additional file 1: Table S1) [16]. In the RIFLE criteria, the baseline creatinine level was defined as the preoperative level. Acute respiratory distress syndrome was defined according to the Berlin definition [17]. Persistent cardiogenic shock was defined as use of inotropic agents, vasopressors, or a mechanical assist device for more than 72 h.

Secondary endpoints were the ICU and hospital lengths of stay, defined as the difference in days between the discharge date and surgery date. Also, the Sequential Organ Failure Assessment (SOFA) score was calculated daily until ICU discharge or for a maximum of 7 days. The initial (0–24 h) and maximum SOFA scores were calculated.

### Statistical analysis

Data were tested for normality by using the Kolmogorov-Smirnov test. Normally distributed data are expressed as means (standard deviation), and non-normally distributed data as median values with interquartile ranges. Continuous variables were compared by using independent *t* tests or the Mann-Whitney *U* test. Categorical variables were compared by means of chi-squared or Fisher’s exact test. For tertile data, continuous variables were compared by analysis of variance (ANOVA) when the distributions were normal and the variances were equivalent; otherwise, they were compared by using the Kruskal-Wallis test. Perioperative VOT data were compared by repeated-measures ANOVA with Bonferroni *post hoc* tests. The study population was divided into tertiles according to the recovery slope on postoperative day 1. The optimal cutoff value for the recovery slope was calculated by applying a receiver operating characteristic curve analysis to test all possible cutoffs that would discriminate composite complications. A cutoff value was chosen from the point in the receiver operating characteristic curve that was the closest to the top left corner of the graph. Associations with clinical outcomes were assessed by using univariable and multivariable linear or logistic regressions. Variables with a *P* value of less than 0.2 were entered into a stepwise linear regression model or a binary logistic analysis with a forward stepwise condition. Multicollinearity was assessed by computing variance inflation factors for all predictors and removing all variables with variance inflation factors of more than 5. A *P* value of less than 0.05 was considered to indicate statistical significance. Statistical analyses were performed by using SPSS software (version 21.0; SPSS Inc., Chicago, IL, USA).

## Results

### Patients

**Fig. 1:**
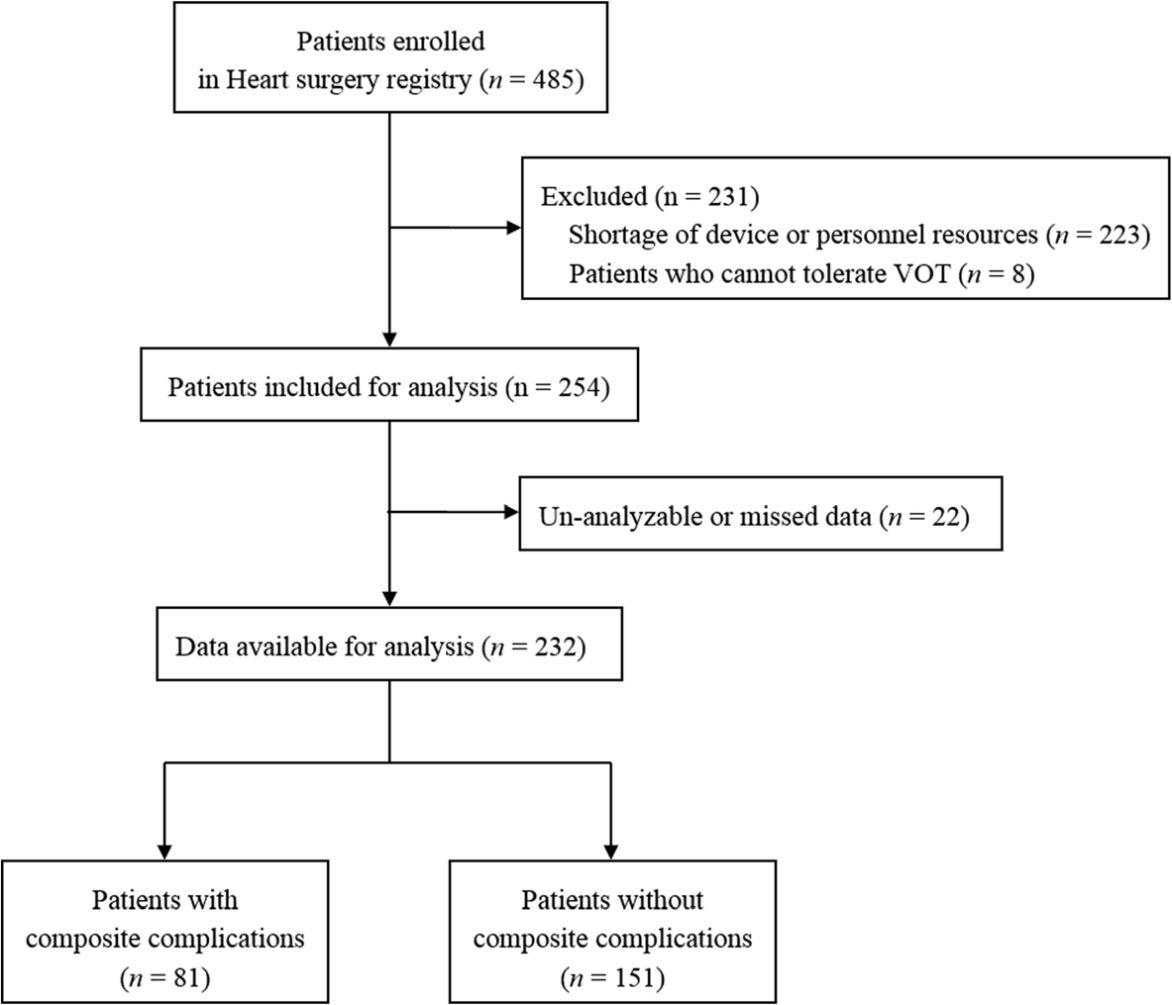
Study flow chart. *VOT* vascular occlusion test

From January 2013 to May 2014, 485 patients were enrolled in the heart registry. Among them, VOTs could not be performed in 231 patients and this was due to the presence of arteriovenous shunts in eight patients and to a shortage of device or personnel resources in 223 patients. Twenty-two patients were not included in the analysis because their NIRS data were either missing or unfit for analysis. Finally, data for 232 patients could be analyzed (Fig. 1). Demographic data for the patients are shown in Table 1. Of the 232 patients, 144 underwent on-pump surgeries and 88 underwent off-pump surgeries.

**Table 1:** Baseline patient, operative characteristics

	All patients	Patients with composite complications	Patients without composite complications	*P* value
	(*n* = 232)	(*n* = 81)	(*n* = 151)	
Demographic characteristics				
Age, years	63 (13)	66 (11)	61 (13)	0.012
Male sex	152 (65.5 %)	53 (65.4 %)	99 (65.6 %)	0.984
Height, cm	163 (9)	163 (10)	163 (8)	0.996
Weight, kg	61 (10)	60 (12)	62 (10)	0.132
Body mass index, kg/m^2^	23.0 (3.3)	22.5 (4.0)	23.3 (2.8)	0.121
Current smokers	42 (18.1 %)	12 (14.8 %)	30 (19.9 %)	0.341
Coexisting conditions				
Diabetes mellitus	61 (26.3 %)	22 (27.2 %)	39 (25.8 %)	0.826
Hypertension	108 (46.6 %)	40 (49.4 %)	68 (45.0 %)	0.527
Stroke	23 (9.9 %)	8 (9.9 %)	15 (9.9 %)	0.989
Dyslipidemia	66 (28.4 %)	26 (32.1 %)	40 (26.5 %)	0.523
Angina	28 (12.1 %)	7 (8.6 %)	21 (13.9 %)	0.241
Myocardiac infarction	8 (3.4 %)	4 (4.9 %)	4 (2.6 %)	0.362
Congestive heart failure	19 (8.2 %)	11 (13.6 %)	8 (5.3 %)	0.028
Liver disease	5 (2.2 %)	2 (2.5 %)	3 (2.0 %)	0.809
Chronic kidney disease	14 (6.1 %)	10 (12.5 %)	4 (2.6 %)	0.003
EuroSCORE II	1.2 (0.8–2.1)	1.7 (1.1–3.2)	1.0 (0.8–1.7)	<0.001
Left ventricle ejection fraction, %	59 (54–64)	58 (55–64)	59 (54–54)	0.617
Preoperative drug therapy				
Angiotensin converting enzyme inhibitor	50 (21.6 %)	17 (21.0 %)	33 (21.9 %)	0.5878
Beta-blocker	71 (31.0 %)	29 (36.3 %)	42 (28.2 %)	0.209
Calcium channel blocker	69 (29.7 %)	19 (23.5 %)	50 (33.1 %)	0.125
Aspirin	111 (47.8 %)	39 (48.1 %)	72 (47.7 %)	0.946
Insulin	19 (8.2 %)	8 (9.9 %)	11 (7.3 %)	0.493
Intraoperative confounders				
Duration of surgery, min	402.3 (112.6)	444.9 (125.7)	379.5 (97.9)	<0.001
Duration of CPB, min^a^	213.3 (88.5)	251.6 (93.1)	181.3 (70.3)	<0.001
Crystalloids, ml/kg	14.0 (8.3 - 22.1)	14.9 (10.4 - 23.6)	13.3 (7.3 - 21.6)	0.259
Colloids, ml/kg	11.6 (1.6 - 17.9)	13.6 (0.0 - 19.8)	10.5 (5.6 - 16.1)	0.320
Packed red blood cell transfusion, units	1 (0–3)	1 (0–5)	1 (0–3)	0.053
Type of procedure				
Valve	103 (44.4 %)	55 (67.9 %)	48 (31.8 %)	<0.001
CABG	96 (41.4 %)	17 (21.0 %)	79 (52.3 %)	<0.001
Valve + CABG	8 (3.4 %)	5 (6.2 %)	3 (2.0 %)	0.096
Aortic valve	47 (20.3 %)	20 (24.7 %)	27 (17.9 %)	0.219
Mitral valve	24 (10.3 %)	15 (18.5 %)	9 (6.0 %)	0.003
Other valves	2 (0.9 %)	0 (0 %)	2 (1.3 %)	0.298
Multivalves	22 (9.5 %)	15 (18.5 %)	7 (4.6 %)	0.001
OPCAB	88 (37.9 %)	12 (14.8 %)	76 (50.3 %)	<0.001
Aorta surgery	10 (4.3 %)	6 (7.4 %)	4 (2.6 %)	0.089
Other procedures	31 (13.4 %)	8 (9.9 %)	23 (15.2 %)	0.253
Reoperation	32 (13.8 %)	22 (26.2 %)	10 (6.8 %)	<0.001

Data are presented as mean (standard deviation), median (interquartile range), or number (percentage).

*EuroSCORE II* European System for Cardiac Operative Risk Evaluation II, *CPB* cardiopulmonary bypass, *CABG* coronary artery bypass graft surgery, *OPCAB* off-pump coronary bypass graft surgery

^a^69 of 81 (85.2 %) patients with composite complications and 75 of 151 (49.7 %) patients without composite complications were exposed to CPB

In the 232 patients, the rate of composite complications was 34.9 %. Patients who developed composite complications had a higher mean age (*P =* 0.012), higher rates of congestive heart failure (*P =* 0.028), and chronic kidney disease (*P =* 0.003) than patients who did not (Table 1). European System for Cardiac Operative Risk Evaluation II (EuroSCORE II) was significantly higher in patients with composite complications than in patients without (*P* < 0.001). There was no significant difference in the gender, weight, body mass index, diabetes, hypertension, or stroke between the patients who developed composite complications and those who did not.

There was no significant difference in the amount of infused fluid or transfused packed red blood cells during the surgery between the patients who developed composite complications and those who did not. However, the duration of surgery was longer in the patients who developed composite complications than those who did not (*P <* 0.001).

### Perioperative VOT values

**Fig. 2:**
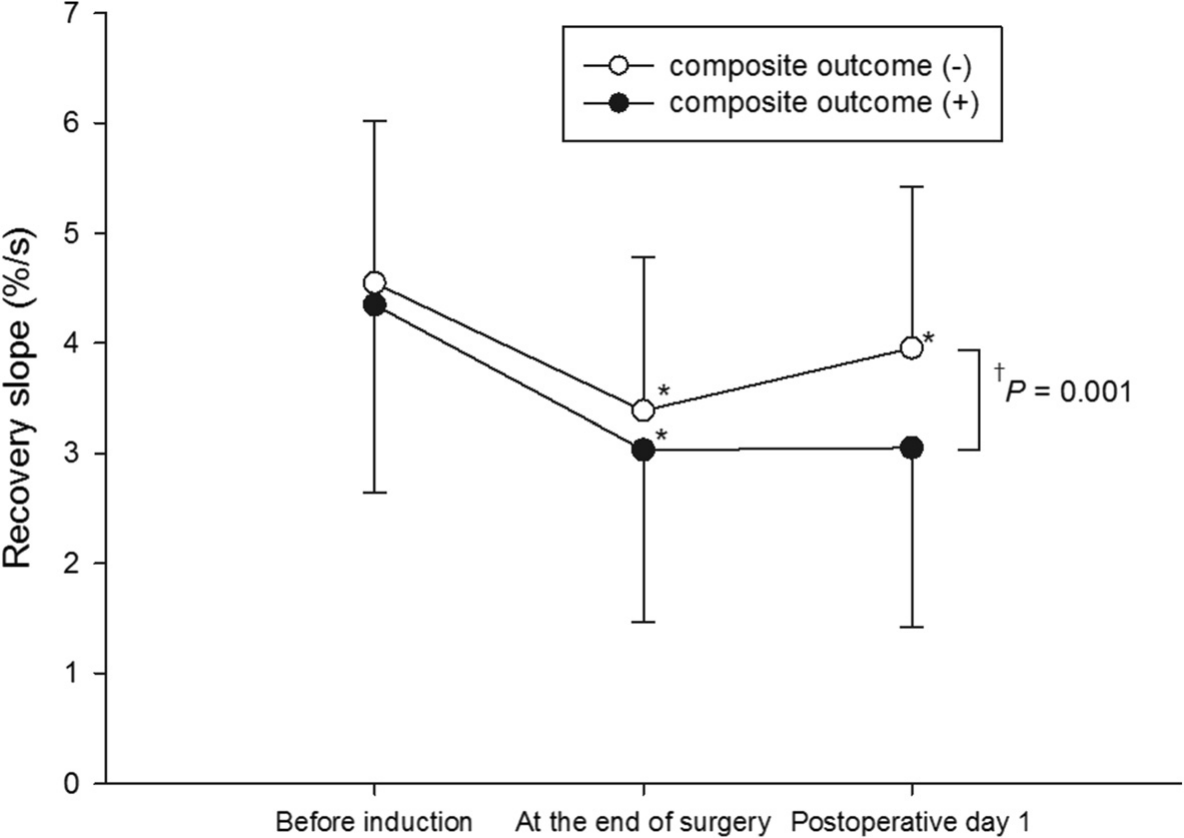
Changes in vascular occlusion test recovery slope during and after the surgery. Values are shown as mean (standard deviation). Asterisks indicate significant differences from the previous measurement (*P <* 0.05). Dagger indicates significant difference in recovery slope between the groups on postoperative day 1

In the overall patients, the recovery slope decreased at the end of surgery (from 4.5 ± 1.6 to 3.3 ± 1.5, *P <* 0.001) and increased on postoperative day 1 (3.7 ± 1.6, *P* < 0.001). Repeated-measures ANOVA indicated that the changes in the recovery slope differed significantly between patients with and without composite complications (*P* = 0.003). In patients without composite complications, the recovery slope decreased at the end of surgery (from 4.5 ± 1.5 to 3.4 ± 1.4 %/s, *P <* 0.001) and increased on postoperative day 1 (4.0 ± 1.5 %/s, *P <* 0.001). In patients with composite complications, the recovery slope decreased at the end of surgery (from 4.3 ± 1.7 to 3.0 ± 1.6 %/s, *P <* 0.001), whereas increase in the recovery slope was not evident on postoperative day 1 (3.1 ± 1.6 %/s, *P* = 0.112; Fig. 2). Bonferroni *post hoc* tests indicated that the recovery slope was significantly lower in patients with composite complications compared with patients without composite complications on postoperative day 1 (*P =* 0.001). The changes in StO_2_ (*P* = 0.277) and occlusion slope (*P* = 0.487) over time were not statistically different in patients with or without composite complications (Table 2).

**Table 2:** Vascular occlusion test parameters by the use of cardiopulmonary bypass

	Total patients			Total patients			Patients undergoing CPB			Patients not undergoing CPB		
	(*n* = 232)			(*n* = 232)			(*n* = 144)			(*n* = 88)		
	Patients undergoing CPB	Patients not undergoing CPB	*P* value	Patients with composite complications	Patients without composite complications	*P* value	Patients with composite complications	Patients without composite complications	*P* value	Patients with composite complications	Patients without composite complications	*P* value
	(*n* = 144)	(*n* = 88)		(*n* = 81)	(*n* = 151)		(*n* = 69)	(*n* = 75)		(*n* = 12)	(*n* = 76)	
Before induction												
Baseline tissue oxygen saturation, %	83.3 (6.2)	84.0 (5.8)	0.425	83.8 (5.9)	83.5 (6.2)	0.683	84.2 (5.8)	82.5 (6.6)	0.139	82.6 (6.3)	84.3 (5.7)	0.266
Occlusion slope, %/min	−9.8 (3.9)	−10.3 (5.2)	0.402	−10.1 (5.1)	−10.0 (4.2)	0.941	−9.9 (4.1)	−9.8 (3.8)	0.825	−10.7 (7.9)	−10.3 (4.5)	0.778
Recovery slope, %/s	4.3 (1.6)	4.7 (1.5)	0.096	4.3 (1.7)	4.5 (1.5)	0.356	4.3 (1.8)	4.3 (1.4)	0.972	4.4 (1.6)	4.7 (1.5)	0.416
At the end of surgery												
Baseline tissue oxygen saturation, %	77.1 (8.1)	77.6 (7.1)	0.631	79.0 (8.0)	76.4 (7.4)	0.061	78.9 (8.3)	75.5 (7.7)	0.021	79.3 (7.1)	77.2 (7.1)	0.261
Occlusion slope, %/min	−9.9 (2.6)	−9.3 (2.9)	0.103	−9.7 (2.6)	−9.6 (2.8)	0.771	−9.7 (2.3)	−10.1 (2.8)	0.484	−9.7 (3.6)	−9.2 (2.8)	0.551
Recovery slope, %/s	3.1 (1.5)	3.4 (1.3)	0.114	3.0 (1.6)	3.4 (1.4)	0.067	2.8 (1.6)	3.4 (1.5)	0.061	3.6 (1.4)	3.4 (1.4)	0.692
Postoperative day 1												
Baseline tissue oxygen saturation, %	85.4 (7.7)	86.5 (6.6)	0.319	86.5 (6.6)	85.7 (7.5)	0.513	86.5 (7.3)	84.3 (8.1)	0.229	86.5 (4.4)	86.5 (7.0)	0.991
Occlusion slope, %/min	−9.0 (2.9)	−10.1 (6.8)	0.175	−8.5 (2.7)	−10.1 (6.0)	0.066	−8.3 (2.8)	−9.6 (2.9)	0.036	−8.9 (2.6)	−10.4 (7.4)	0.482
Recovery slope, %/s	3.6 (1.7)	3.7 (1.4)	0.723	3.1 (1.6)	4.0 (1.5)	0.001	2.9 (1.7)	4.2 (1.6)	<0.001	3.4 (1.6)	3.8 (1.4)	0.373

Data are presented as mean (standard deviation). *CPB* cardiopulmonary bypass

In patients with CPB, difference of VOT recovery slope was more prominent on postoperative day 1 (2.9 ± 1.7 versus 4.2 ± 1.6 %/s, *P* < 0.001, Table 2). In patients without CPB, recovery slope on postoperative day 1 was lower in patient with composite complications without statistical significance 1 (3.4 ± 1.6 versus 3.8 ± 1.4 %/s, *P* = 0.373).

### Clinical outcomes and VOT values

At the end of surgery, neither conventional hemodynamic measurements, such as blood pressure, cardiac index, and mixed venous saturation, nor VOT values differed significantly between patients with and without composite complications (Table 3). However, on postoperative day 1, the VOT recovery slope was significantly lower in the patients with composite complications than in those without (3.1 ± 1.6 versus 4.0 ± 1.5 %/s, *P* = 0.001). Hemodynamic parameters did not differ between groups, with the exception of central venous pressure, which was higher in the patients with composite complications. At both time points, the use of inotropes and vasopressors was higher in patients with composite complications than in patients without (Table 3).

**Table 3:** Hemodynamic and vascular occlusion test parameters

Measurements	Patients with composite complications	Patients without composite complications	*P* value
	(*n* = 81)	(*n* = 151)	
At the end of surgery			
Heart rate	80 (70–90)	76 (66–84)	0.074
SpO_2_, %	100 (99–100)	100 (100–100)	0.111
Mean arterial pressure, mm Hg	68 (64–82)	74 (67–78)	0.089
Central venous pressure, mm Hg	10 (6–12)	7 (5–10)	0.002
Cardiac index, l/min per m^2^	2.5 (0.9)	2.4 (0.6)	0.311
Mixed venous saturation, %	70 (7)	72 (6)	0.051
Temperature, °C	35.8 (0.6)	35.9 (0.6)	0.228
Use of inotropes	32 (39.5 %)	20 (13.2 %)	<0.001
Use of vasopressors	35 (43.2 %)	45 (29.8 %)	0.041
Lactate, mmol/l	2.0 (1.2–3.2)	1.5 (1.0–2.1)	0.001
Tissue oxygen saturation, %	78.4 (7.9)	76.4 (8.1)	0.061
VOT occlusion slope, %/min	−9.7 (2.6)	−9.6 (2.8)	0.771
VOT recovery slope, %/s	3.0 (1.6)	3.4 (1.4)	0.067
First postoperative day			
Heart rate	82 (76–92)	78 (72–86)	0.005
SpO_2_, %	100 (99 – 100)	100 (100–100)	0.669
Mean arterial pressure, mm Hg	74 (68–78)	77 (68–80)	0.310
Central venous pressure, mm Hg	8 (7–10)	7 (5–9)	<0.001
Cardiac index, l/min per m^2^	2.7 (0.6)	2.7 (0.5)	0.857
Temperature, °C	36.7 (0.7)	36.8 (0.6)	0.115
Use of inotropes	60 (74.1 %)	88 (58.7 %)	0.020
Use of vasopressors	27 (33.3 %)	19 (12.6 %)	<0.001
Use of midazolam	20 (24.7 %)	7 (4.6 %)	<0.001
Use of remifentanil	38 (46.9 %)	29 (19.2 %)	<0.001
Tissue oxygen saturation, %	86.5 (6.6)	85.7 (7.5)	0.513
VOT occlusion slope, %/min	−8.5 (2.7)	−10.1 (6.0)	0.066
VOT recovery slope, %/s	3.1 (1.6)	4.0 (1.5)	0.001

Data are presented as mean (standard deviation), median (interquartile range), or number (percentage)

*SpO*_*2*_ peripheral capillary oxygen saturation, *VOT* vascular occlusion test

**Table 4:** Clinical outcomes by the tertile according to the recovery slope on postoperative day 1

	Total^a^	Total^b^	Lowest tertile	Middle tertile	Highest tertile	*P* value
	(*n* = 232)	(*n* = 173)	(*n* = 58)	(*n* = 57)	(*n* = 58)	
Composite complications	81 (34.9 %)	58 (33.5 %)	28 (48.3 %)	18 (31.6 %)	12 (20.7 %)	0.007
In-hospital mortality	6 (2.6 %)	5 (2.9 %)	2 (3.4 %)	2 (3.5 %)	1 (1.7 %)	0.809
Myocardial infarction	2 (0.9 %)	2 (1.2 %)	0 (0.0 %)	1 (1.8 %)	1 (1.7 %)	0.600
Acute kidney injury						
RIFLE Risk category	56 (24.1 %)	38 (22.0 %)	16 (27.6 %)	13 (22.8 %)	9 (15.5 %)	0.287
RIFLE Injury category	28 (12.1 %)	20 (11.6 %)	10 (17.2 %)	6 (10.5 %)	4 (6.9 %)	0.210
RIFLE Failure category	25 (10.8 %)	20 (11.6 %)	12 (20.7 %)	5 (8.8 %)	3 (5.2 %)	0.024
Renal replacement therapy	15 (6.5 %)	14 (8.1 %)	8 (13.8 %)	4 (7.0 %)	2 (3.4 %)	0.116
Acute respiratory distress syndrome	7 (3.0 %)	7 (4.0 %)	3 (5.2 %)	3 (5.3 %)	1 (1.7 %)	0.545
Persistent cardiogenic shock	32 (13.8 %)	24 (13.9 %)	15 (25.9 %)	7 (12.3 %)	2 (3.4 %)	0.002
Initial SOFA	9 (8–11)	5 (3–7)	6 (3–9)	5 (3–8)	3 (4–9)	0.005
Maximum SOFA	12 (10–14)	6 (4–9)	7 (4–10)	7 (4–9)	5 (3–7)	0.001
Mechanical ventilation-free days, days 1 to 28	27.2 (27.0–27.4)	27.2 (27.0–27.4)	27.1 (26.2–27.3)	27.3 (27.0–27.4)	27.3 (27.2–27.5)	0.004
Intensive care unit length of stay, days	4 (3–6)	4 (3–6)	5 (3–8)	3 (2–6)	3 (2–5)	0.004
Hospital length of stay, days	11 (11–18)	11 (8–18)	14 (10–29)	12 (9–17)	9 (8–15)	0.002

Data are presented as number (percentage) or median (interquartile range)

*RIFLE* Risk, Injury, Failure, Loss and End-stage Renal Disease, *SOFA* Sequential Organ Failure Assessment

^a^Total patients with composite complications data

^b^Total patients with postoperative day 1 recovery slope data

**Fig. 3:**
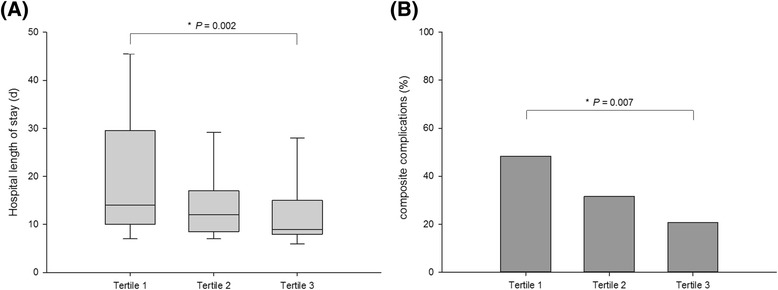
**a** Hospital length of stay. **b** Rate of composite complications stratified by tertiles of recovery slope on postoperative day 1. The boxes indicate the first quartile (bottom line), the median (central line) and the third quartile (upper line). The whiskers represent whiskers represent 10 %/90 % quantiles. Lowest tertile < 2.9 %/s, middle tertile 2.9–4.3 %/s, and highest tertile > 4.3 %/s

The study population was divided into tertiles according to the recovery slope on postoperative day 1: lowest tertile of less than 2.9 %/s, middle tertile 2.9–4.3 %/s, and highest tertile of more than 4.3 %/s (Table 4). There were significant differences among the tertiles in the ICU (*P =* 0.004) and hospital (*P =* 0.002) length of stay and the rate of composite complications (*P =* 0.007; Fig. 3). A significantly increased risk of composite complications was observed for the lowest tertile (odds ratio (OR) = 3.578, 95 % confidence interval (CI) = 1.579–8.106, *P =* 0.002) but not in the middle tertile (OR = 1.769, 95 % CI = 0.759–4.122, *P =* 0.186) compared with the highest tertile. Patients in the lowest tertile of the recovery slope on postoperative day 1 showed 5-day-longer hospital length of stay than the highest tertile (14 (10.0–29.5) versus 9 (8.0–15.0), *P =* 0.002)). Remifentanil and midazolam usage on postoperative day 1 were not different between tertiles.

Duration of surgery (*r* = −0.180, *P =* 0.017) and lactate level at the end of surgery (*r* = −0.177, *P =* 0.012) were significantly correlated with recovery slope on postoperative day 1. No significant correlations were found between postoperative recovery slope and other intraoperative parameters, such as heart rate, arterial pressure, cardiac index, and postoperative day 1 troponin I level. EuroSCORE II (*r* = −0.205, *P =* 0.007) was significantly correlated with recovery slope on postoperative day 1.

Univariable logistic regression analyses were performed, and age, congestive heart failure, chronic kidney disease, use of the CPB, valvular surgery, EuroSCORE II, use of vasopressor at the end of surgery, lactate at the end of surgery, central venous pressure at the end of surgery, and VOT recovery slope on postoperative day 1 were associated with composite complications (*P <* 0.05). Multivariable logistic regression analysis indicated that age, chronic kidney disease, valvular surgery, and VOT recovery slope on postoperative day 1 were associated with the composite complications (Model 1; Additional file 2: Table S2). Univariable linear regression analyses showed that age, congestive heart failure, stroke, chronic kidney disease, use of the CPB, central venous pressure at the end of surgery, and VOT recovery slope on postoperative day 1 were associated with hospital length of stay (*P <* 0.05). Multivariable analysis indicated that body mass index, chronic kidney disease, valvular surgery, and VOT recovery slope on postoperative day 1 were associated with hospital length of stay (Model 1; Additional file 3: Table S3). According to the receiver operating characteristic curve for the recovery slope on postoperative day 1, the cutoff value for predicting composite complications was 3.2 %/s with a sensitivity of 58.1 % and a specificity of 68.6 % (area under the curve 0.668, 95 % CI 0.583–0.753, *P* < 0.001, Additional file 4: Figure S1).

## Discussion

In this study, the VOT recovery slope decreased at the end of surgery in cardiac surgery patients. The VOT recovery slope recovered on postoperative day 1 in patients without composite complications but did not in patient with composite complications. The lowest tertile of the recovery slope on postoperative 1 showed a three- to fourfold higher risk of composite complications and 5-day-longer hospital length of stay than the highest tertile. VOT recovery slope on postoperative day 1 was independently related with composite complications.

Previous studies have reported that the recovery slope was associated with clinical outcomes in septic shock patients [10, 12]. Likewise, in the current study, recovery slope on postoperative day 1 was associated with composite complications in cardiac surgery patients. Also, hospital length of stay was longest in lowest tertile of recovery slope. Few studies have evaluated the relationship between dynamic StO_2_ parameters and clinical outcomes in patients undergoing cardiac surgery. In a recent study by Morel et al. [18], recovery slope was not correlated with ICU length of stay or SOFA score in cardiac surgery (Additional file 5: Table S4). However, they enrolled fewer patients (*n* = 40) than this study (*n* = 232). Forty patients might have been too few to evaluate the effects on clinical outcomes. Moreover, the CPB time of that study was 128 min, less than in this study (193 ± 104 min). This suggests that the inflammatory reaction may have been smaller in the study by Morel et al.

The VOT recovery slope has been used as an index of microvascular function, reflecting microvascular reperfusion and reactivity [11, 19, 20]. It was correlated with peripheral perfusion parameters in critically ill patients but was independent of the conventional hemodynamic parameters [9]. Especially in sepsis, endothelial cell function and microvascular reactivity are altered, with arteriovenous shunting and closed capillaries [12, 21]. Consequently, tissue perfusion could be impaired even with normal arterial pressure and mixed venous oxygenation [22, 23]. Previous research has shown that dynamic StO_2_ parameters were superior to conventional parameters in predicting prognosis in patients with septic [10, 19]. This is consistent with our results, in which the VOT recovery slope was associated with composite complications whereas conventional hemodynamic parameters, such as arterial pressure or cardiac index, did not show an association on postoperative day 1 (Table 3). However, there were group differences in the use of inotropes or vasopressors. These results can be explained by the fact that arterial pressure and the cardiac index are usually the targets of hemodynamic management. Consequently, there were no differences in these variables, but there were differences in the therapies, such as the use of inotropes or vasopressors to reach the target blood pressure or cardiac index.

Cardiac surgery provokes a vigorous inflammatory response and can induce systemic inflammatory response syndrome (SIRS) [13]. Surgical trauma, blood loss, transfusion, hypothermia, hypoperfusion, and CPB may be causes of the inflammatory reaction. The vascular endothelium plays a pivotal role in this inflammatory reaction and microcirculatory derangement during cardiac surgery [13]. As SIRS impairs the microcirculation and ultimately can lead to multiorgan failure, we hypothesized and showed in this study that a perioperative decrease in microcirculatory function may be associated with adverse clinical outcomes in cardiac surgery patients. Several studies have shown that VOT recovery slope decreases during CPB but returns to the baseline value postoperatively [18, 24]. This perioperative change in the recovery slope was also seen in the present study. Moreover, in the present study, the impaired VOT recovery slope on postoperative day 1 was independently related to composite complications, suggesting that microvascular reactivity is impaired during cardiac surgery and furthermore that this is associated with poor clinical outcomes, as observed in sepsis. A decreased recovery slope represents a deficit in the capacity to recruit microvessels in response to a hypoxic stimulus, which might be associated with local tissue inflammation, impaired oxygen extraction, and ultimately organ dysfunction after cardiac surgery [10, 12].

The effects of CPB on microcirculatory alterations and inflammation are well known [8, 13, 24]. In this study, we compared the patient data according to the use of CPB (Table 2). In patients with CPB, the difference in the recovery slope between patients with and without complications was more prominent on postoperative day 1. However, the VOT variables did not differ in patients with and without CPB. In addition, the use of CPB did not show a significant association with composite complications in the multivariable model. A better evaluation of the effect of CPB might be achieved by controlling possible confounding factors such as the type of surgery, duration of CPB, and temperature.

Our results demonstrated that postoperative day 1 recovery slope was related to duration of surgery and lactate level at the end of surgery. Hyperlactemia, a well-known marker of tissue hypoperfusion, is frequently observed during and after cardiac surgery [25]. High lactate level at the end of surgery may be the result of intraoperative occult tissue hypoperfusion and associated with postoperative complications [26]. Likewise, in the present study, lactate level was higher in patients with composite complications than in patients without (Table 3).

The VOT occlusion slope reflects oxygen metabolism in the tissue; thus, a low occlusion slope indicates impaired regional perfusion distribution, lower metabolic rate, or impairment in oxygen utilization by mitochondria [11, 27]. Several studies have reported that the occlusion slope is lower in septic shock patients [28, 29], and the prehospital occlusion slope was associated with the need for lifesaving intervention in trauma patients [27]. In the present study, the occlusion slope over time did not differ significantly between patients with and without composite complications. However, a decreasing trend was seen in patients with composite complications, and the occlusion slope on postoperative day 1 was lower in patients with composite complications, although this lacked statistical significance (−8.5 ± 2.7 versus −10.1 ± 6.0 %/min, *P* = 0.066, Table 2).

VOT-derived occlusion and recovery slopes provide semicontinuous measurements of microvascular reactivity, which may be an important target for therapy in surgery [10, 11]. Pathological changes in tissue microcirculation reactivity, as indicated by a low recovery slope, may provide information regarding the prognosis in patients undergoing cardiac surgery. However, to date, what should be done for patients who show impaired microcirculatory reactivity is unknown. Further research is needed on this topic.

This study had several limitations. First, it was a single-center study and enrolled a heterogeneous cardiac surgery population. Moreover, in our center, coronary artery bypass graft surgery is routinely performed without CPB; indeed, only eight coronary artery bypass graft surgery cases were performed with CPB. Thus, our results do not reflect the on-pump coronary artery bypass graft surgery scenario. Second, the VOT itself has not been standardized with regard to the site of measurement, ischemic threshold, or time interval between tests [30]. Also, the VOT occlusion or recovery slope does not always show clear linearity and this is due to technical issues in sampling or voluntary thenar muscle activity [11]. Third, VOT was not performed serially in the ICU. Thus, the question of when the recovery slope is restored completely to preoperative levels was not investigated in the current study. Fourth, use of vasopressors might confound VOT results. In this study, vasopressors were used more frequently in patients with composite complications than in those without. Although vasopressors are used to increase mean arterial pressure and perfusion pressure, high-dose vasopressor may cause various peripheral hypoperfusion conditions [31]. In spite of more frequent use of inotropics and vasopressors, cardiac output and blood pressure were not different between the patients with and those without complications. It may suggest that the use of inotropics and vasopressors may improve hemodynamic status but not clinical outcomes. Fifth, in this study, anesthesia was maintained with remifentanil-propofol continuous infusion, which may increase muscle blood flow [32]. Thus, the results may differ with inhalational agent-based anesthesia.

## Conclusions

Microvascular reactivity, assessed by VOT recovery slope, decreases during cardiac surgery and recovers on postoperative day 1. Patients with lower recovery slopes on postoperative day 1 showed higher complication rates and longer hospital length of stay. Postoperative restoration of microvascular reactivity is related to clinical outcomes in cardiac surgery patients.

## Key message

- Microvascular reactivity, assessed by VOT recovery slope, decreased during cardiac surgery.
- Microvascular reactivity largely recovered on postoperative day 1 in patients without composite complications, but this restoration was attenuated in patients with composite complications.
- The lowest tertile of the recovery slope on postoperative 1 showed a higher composite complication rate and longer hospital length of stay than the highest tertile.
- Microvascular reactivity on postoperative day 1 was independently related with composite complications.

## Additional files

Additional file 1: Table S1. RIFLE classification for acute kidney injury. (DOCX 16 kb) Additional file 2: Table S2. Independent contributors to composite complications. (DOCX 17 kb) Additional file 3: Table S3. Independent contributors to the hospital length of stay. (DOCX 17 kb) Additional file 4: Figure S1. Receiver operating characteristic curve for the recovery slope on postoperative day 1 to discriminate the composite complications. According to the receiver operating characteristic curve, the cut-off point that yielded the maximal sensitivity and specificity for predicting composite complications was 3.2 %/s, and the sensitivity and specificity using the cut-off value were 58.1 % and 68.6 %, respectively (area under the curve 0.668, 95 % CI 0.583-0.753, *P* < 0.001). (TIFF 44 kb) Additional file 5: Table S4. Vascular occlusion test parameters by the ICU length of stay. (DOCX 16 kb)

## Abbreviations

ANOVA: Analysis of variance
CI: Confidence interval
CPB: Cardiopulmonary bypass
EuroSCORE II: European System for Cardiac Operative Risk Evaluation II
ICU: Intensive care unit
NIRS: Near-infrared spectroscopy
OR: Odds ratio
RIFLE: Risk, Injury, Failure, Loss, and End-Stage Kidney Disease
SIRS: Systemic inflammatory response syndrome
SOFA: Sequential Organ Failure Assessment
StO_2_: Tissue oxygen saturation
VOT: Vascular occlusion test
