# Supplementary material for: Microvascular reactivity and clinical outcomes in cardiac surgery
Source: Crit Care. 2015 Sep 4;19(1):316. doi: 10.1186/s13054-015-1025-3 (PMC4560090; doi:10.1186/s13054-015-1025-3)
Supplement: Additional file 1: Table S1. — RIFLE classification for acute kidney injury. (DOCX 16 kb) [file 13054_2015_1025_MOESM1_ESM.docx]

**Additional file 1: Table S1.** RIFLE Classification for Acute Kidney Injury

| RIFLE Risk category | Increase in serum creatinine value ≥150% from baseline |
| --- | --- |
| RIFLE Injury category | Increase in serum creatinine value ≥200% from baseline |
| RIFLE Failure category | Increase in serum creatinine value ≥300% from baseline or an absolute serum creatinine ≥4 mg/dL with an increase ≥ 0.5 mg/dL from baseline. Any patient who received acute dialysis is categorized in this category. |

The absolute creatinine rise and percentage change from baseline in the 48 h after surgery.

RIFLE = Risk, Injury, Failure, Loss and End-stage Renal Disease.
