# Supplementary material for: Microvascular reactivity and clinical outcomes in cardiac surgery
Source: Crit Care. 2015 Sep 4;19(1):316. doi: 10.1186/s13054-015-1025-3 (PMC4560090; doi:10.1186/s13054-015-1025-3)
Supplement: Additional file 2: Table S2. — Independent contributors to composite complications. (DOCX 17 kb) [file 13054_2015_1025_MOESM2_ESM.docx]

**Additional file 2: Table S2.** Independent contributors to composite complications

|  | Univariable analysis | | | Multivariable analysis | | | | | |
| --- | --- | --- | --- | --- | --- | --- | --- | --- | --- |
|  |  | | | Model 1*^a^* | | | Model 2*^b^* | | |
|  | OR | 95% CI | *P* Value | OR | 95% CI | *P* Value | OR | 95% CI | *P* Value |
| Age, yr | 1.030 | 1.006 - 1.055 | 0.014 | 1.036 | 1.000 -1.074 | 0.049 | 1.048 | 1.007 - 1.090 | 0.021 |
| Male sex | 1.006 | 0.570 - 1.775 | 0.984 |  |  |  |  |  |  |
| Body mass index, kg/m^2^ | 0.927 | 0.850 - 1.011 | 0.087 |  |  |  |  |  |  |
| Congestive heart failure | 2.809 | 1.081 - 7.296 | 0.034 |  |  |  |  |  |  |
| Diabetes mellitus | 1.071 | 0.582 - 1.971 | 0.826 |  |  |  |  |  |  |
| Hypertension | 1.191 | 0.693 - 2.045 | 0.527 |  |  |  |  |  |  |
| Stroke | 0.994 | 0.402 - 2.454 | 0.989 |  |  |  |  |  |  |
| Chronic kidney disease | 5.250 | 1.591 - 17.326 | 0.006 | 8.658 | 2.026 – 36.988 | 0.004 | 10.224 | 2.279 - 45.870 | 0.002 |
| Use of CPB | 4.131 | 2.216 - 7.701 | < 0.001 |  |  |  |  |  |  |
| Valvular surgery | 4.539 | 2.545 - 9.097 | < 0.001 | 7.253 | 3.420 – 15.383 | < 0001 | 7.883 | 3.433 - 17.874 | <0.001 |
| PRBC transfusion, u | 0.998 | 0.982 - 1.014 | 0.772 |  |  |  |  |  |  |
| EuroSCORE II | 1.618 | 1.293 - 2.026 | < 0.001 |  |  |  |  |  |  |
| Use of vasopressor at the end of surgery | 1.792 | 1.023 - 3.141 | 0.042 |  |  |  |  |  |  |
| Lactate at the end of surgery | 1.362 | 1.120 - 1.655 | 0.002 |  |  |  | 1.311 | 1.069 - 1.609 | 0.009 |
| CVP at the end of surgery | 1.124 | 1.039 - 1.215 | 0004 |  |  |  |  |  |  |
| VOT recovery slope on postoperative day 1, %/s | 0.684 | 0.552 - 0.847 | 0.001 | 0.742 | 0.584 – 0.943 | 0.015 | 0.763 | 0.591 - 0.985 | 0.038 |

*^a^*Model 1: adjusted for age, body mass index, congestive heart failure, chronic kidney disease, use of CPB, valvular surgery, use of vasopressor at the end of surgery, CVP at the end of surgery. *^b^*Model 2: adjusted for Model 1 variables and additionl covariables: (i) EuroSCORE II and (ii) lactate at the end of surgery. OR, odds ratio; CI, confidence interval; CPB, cardiopulmonary bypass; PRBC, packed red blood cell; EuroSCORE, European System for Cardiac Operative Risk Evaluation; CVP, central venous pressure; VOT, vascular occlusion test.
