# Supplementary material for: Microvascular reactivity and clinical outcomes in cardiac surgery
Source: Crit Care. 2015 Sep 4;19(1):316. doi: 10.1186/s13054-015-1025-3 (PMC4560090; doi:10.1186/s13054-015-1025-3)
Supplement: Additional file 3: Table S3. — Independent contributors to the hospital length of stay. (DOCX 17 kb) [file 13054_2015_1025_MOESM3_ESM.docx]

**Additional file 3: Table S3.** Independent contributors to the hospital length of stay

|  | Univariable analysis | | | Multivariable analysis | | | | | |
| --- | --- | --- | --- | --- | --- | --- | --- | --- | --- |
|  |  | | | Model 1*^a^* | | | Model 2*^b^* | | |
|  | B | 95% CI | *P* Value | B | 95% CI | *P* Value | B | 95% CI | *P* Value |
| Age, yr | 0.221 | 0.011 - 0.430 | 0.039 |  |  |  |  |  |  |
| Male sex | -2.868 | -8.462 - 2.727 | 0.314 |  |  |  |  |  |  |
| Body mass index, kg/m^2^ | -0.177 | -0.440 - 0.087 | 0.188 | -0.600 | -1.179 - -0.002 | 0.042 |  |  |  |
| Congestive heart failure | 11.303 | 1.695 - 20.910 | 0.021 |  |  |  |  |  |  |
| Diabetes mellitus | 5.127 | -0.890 - 11.144 | 0.095 |  |  |  |  |  |  |
| Hypertension | 2.008 | -3.328 - 7.345 | 0.459 |  |  |  |  |  |  |
| Stroke | 13.137 | 4.384 - 21.890 | 0.003 |  |  |  |  |  |  |
| Chronic kidney disease | 19.562 | 8.658 - 30.466 | < 0.001 | 17.320 | 9.673 – 24.968 | < 0.001 | 13.284 | 5.680 - 20.887 | 0.001 |
| Use of CPB | 5.369 | 0.003 - 10.736 | 0.049 |  |  |  |  |  |  |
| Valvular surgery | 3.909 | -1.431 - 9.249 | 0.151 | 7.520 | 3.726 – 11.314 | < 0.001 | 5.206 | 1.605 - 8.807 | 0.005 |
| PRBC transfusion, u | -0.002 | -0.137 - 0.132 | 0.971 |  |  |  |  |  |  |
| EuroSCORE II | 3.621 | 2.275 - 4.967 | < 0.001 |  |  |  | 1.863 | 0.764 - 2.961 | 0.001 |
| Use of vasopressor at the end of surgery | 2.551 | -3.047 - 8.148 | 0.370 |  |  |  |  |  |  |
| Lactate at the end of surgery | 2.580 | 1.277 - 3.882 | < 0.001 |  |  |  | 2.046 | 1.107 - 2.985 | < 0.001 |
| CVP at the end of surgery | 0.935 | 0.195 - 1.676 | 0.014 |  |  |  |  |  |  |
| VOT recovery slope on postoperative day 1, %/s | -1.798 | -3.099 - 0.497 | 0.007 | -1.276 | -2.440 - -0.112 | 0.032 |  |  |  |

*^a^*Model 1: adjusted for age, body mass index, congestive heart failure, diabetes mellitus, stroke, chronic kidney disease, use of CPB, valvular surgery, CVP at the end of surgery. *^b^*Model 2: adjusted for Model1 variables and additionl covariables: (i) EuroSCORE II and (ii) lactate at the end of surgery. B, Regression coefficient; CI, confidence interval; CPB, cardiopulmonary bypass; PRBC, packed red blood cell; EuroSCORE, European System for Cardiac Operative Risk Evaluation; CVP, central venous pressure; VOT, vascular occlusion test
