# Supplementary figures and images for: Microvascular reactivity and clinical outcomes in cardiac surgery
Source: Crit Care. 2015 Sep 4;19(1):316. doi: 10.1186/s13054-015-1025-3 (PMC4560090; doi:10.1186/s13054-015-1025-3)

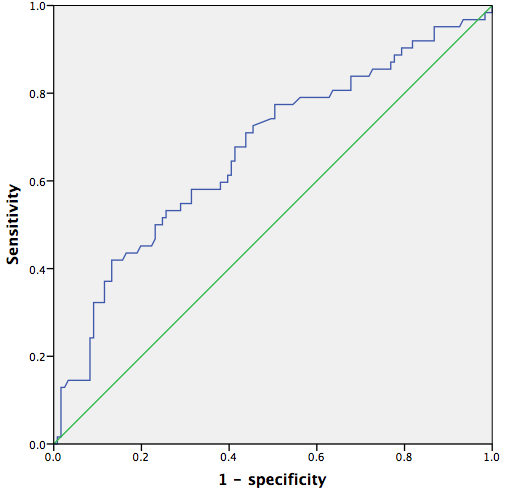

Supplement: Additional file 4: Figure S1. — Receiver operating characteristic curve for the recovery slope on postoperative day 1 to discriminate the composite complications. According to the receiver operating characteristic curve, the cut-off point that yielded the maximal sensitivity and specificity for predicting composite complications was 3.2 %/s, and the sensitivity and specificity using the cut-off value were 58.1 % and 68.6 %, respectively (area under the curve 0.668, 95 % CI 0.583-0.753, P < 0.001). (TIFF 44 kb) [file 13054_2015_1025_MOESM4_ESM.tif]
