# Supplementary material for: Microvascular reactivity and clinical outcomes in cardiac surgery
Source: Crit Care. 2015 Sep 4;19(1):316. doi: 10.1186/s13054-015-1025-3 (PMC4560090; doi:10.1186/s13054-015-1025-3)
Supplement: Additional file 5: Table S4. — Vascular occlusion test parameters by the ICU length of stay. (DOCX 16 kb) [file 13054_2015_1025_MOESM5_ESM.docx]

**Additional file 5: Table S4.** Vascular occlusion test parameters by the ICU length of stay

|  | Patients with  ICU length of stay ≤ 4  (*n* = 141) | Patients with ICU length of stay > 4 (*n* = 91) | *P* Value |
| --- | --- | --- | --- |
| **Vascular occlusion test parameters** | | | |
| Before induction | | | |
| Baseline tissue oxygen saturation, % | 84.2 (5.6) | 82.7 (6.6) | 0.066 |
| Occlusion slope, %/min | -10.1 (4.6) | -10.0 (4.4) | 0.840 |
| Recovery slope, %/s | 4.5 (1.5) | 4.4 (1.7) | 0.746 |
| At the end of surgery | | | |
| Baseline tissue oxygen saturation, % | 76.5 (7.5) | 78.6 (7.9) | 0.060 |
| Occlusion slope, %/min | -9.9 (2.8) | -9.3 (2.6) | 0.151 |
| Recovery slope, %/s | 3.5 (1.4) | 2.9 (1.5) | 0.008 |
| On postoperative day 1 | | | |
| Baseline tissue oxygen saturation, % | 85.8 (7.5) | 86.2 (6.8) | 0.738 |
| Occlusion slope, %/min | -9.8 (6.3) | -9.1 (2.9) | 0.414 |
| Recovery slope, %/s | 3.9 (1.5) | 3.2 (1.6) | 0.002 |
| **Postoperative complications** |  |  |  |
| Composite complications | 25 (17.7%) | 56 (61.5%) | < 0.001 |
| Myocardial infarction | 2 (1.4%) | 0 (0.0%) | 0.521 |
| Acute kidney injury | 19 (13.5%) | 45 (49.5%) | < 0.001 |
| Acute respiratory distress syndrome | 0 (0.0%) | 7 (7.7%) | 0.001 |
| Persistent cardiovascular shock | 5 (3.5%) | 27 (29.7%) | < 0.001 |
| Mechanical ventilation free-days, days 1 to 28 | 27.3 (27.2-27.5) | 26.7 (24.3-27.2) | < 0.001 |
| Initial SOFA | 4 (2-6) | 8 (6-10) | < 0.001 |
| Maximum SOFA | 4 (3-6) | 9 (7-12) | < 0.001 |

Data are presented as mean (SD), n (%) or median (IQR).
